# Multi-level determinants of breast cancer screening among Malay-Muslim women in Singapore: a sequential mixed-methods study

**DOI:** 10.1186/s12905-022-01972-y

**Published:** 2022-09-19

**Authors:** Su-Ann Goh, Jeong Kyu Lee, Wei Yan Seh, Elaine Qiao Ying Ho, Mikael Hartman, Cynthia Chou, Mee Lian Wong

**Affiliations:** 1 Saw Swee Hock School of Public Health, National University of Singapore and National University Health System, Tahir Foundation Building, 12 Science Drive 2 #10-01, Singapore, 117549 Singapore; 2 Department of Health and Exercise Science, University of Oklahoma, Oklahoma, USA; 3 Department of Surgery, Yong Loo Lin School of Medicine, National University of Singapore and National University Health System, Singapore, Singapore; 4 Department of Surgery, National University Hospital and National University Health System, Singapore, Singapore; 5 Department of Anthropology, The University of Iowa, Iowa, USA

**Keywords:** Breast cancer screening, Malay-Muslim women, Mixed-methods, Barriers, Facilitators, Southeast Asia

## Abstract

**Objective:**

Ethnic disparity persists despite equal access to health care in Singapore, with Malay-Muslim women having the lowest mammogram uptake rate and highest breast cancer mortality rate. We sought to understand barriers to and facilitators for mammogram uptake in this community.

**Methods:**

We used a sequential mixed-methods design to first explore reasons for screening and not screening for breast cancer, then determine factors associated with screening and regular screening in a survey. We used maximum variation sampling for semi-structured in-depth interviews to select screeners and non-screeners of diverse ages and educational levels. Twenty-three Malay-Muslim women aged 40–69 years old were interviewed. Themes were categorized using thematic analysis. For the survey, we applied the Health Belief Model, Social Ecological Model, as well as themes from the interviews and findings from previous studies on factors influencing screening in Muslim women to guide questionnaire design. We surveyed 271 Malay-Muslim women aged 50–69 years old in a nationally representative sample. Multivariable logistic regression was used to determine factors associated with ever gone for mammogram and regular mammogram uptake.

**Results:**

Through in-depth-interviews, we found perceived benefits of saving lives and breasts from early detection, reminders from doctors and husbands, symptoms, perceived test from God, and personal responsibility to care for one’s health facilitated screening. Barriers were perceived low susceptibility, inconvenience, cost, negative psychological effects, misinformation on mammogram triggering cancer cells, religious beliefs, perceived negative outcomes from mammography and distrust of doctor. From the survey, we found cues from health care professionals and needing symptoms before deciding to go for mammogram to be significantly associated with ever gone for mammogram and regular mammogram. Factors associated with ever gone for mammogram only included age, perceived benefits of saving lives from early detection, perceived importance of mammogram, Punishing Allah Reappraisal, and modesty. Factors associated with regular mammogram only included household income, perceived structural barriers to screening and perceived susceptibility to breast cancer.

**Conclusions:**

Mammogram uptake is affected by multiple levels of influence. Interventions to promote screening should be designed with multiple stakeholders including doctors, religious leaders and women who had attended screening.

**Supplementary Information:**

The online version contains supplementary material available at 10.1186/s12905-022-01972-y.

## Background

Female breast cancer is the most diagnosed cancer and leading female cancer-associated death globally [1]. In some high-income countries, 5-year survival rates above 90% has been reported at the earliest stages of breast cancer [2, 3]. As breast cancer is highly treatable if detected early, timely screening is key to reducing breast cancer mortality.

Singapore is one such country with high incidence and high 5-year survival rate [2]. Singapore has implemented a national screening program since 2002 [4], made screening facilities available nationwide [5], and reduced out-of-pocket cost of screening through subsidies and through direct deduction from the national medical savings scheme, MediSave [6]. These efforts to ensure equal access however, have not translated to equal uptake across its multi-ethnic population. Singapore is home to 4.04 million residents mostly from the three major Asian ethnic groups, Chinese (74.3%), Malays (13.5%), and Indians (9.0%)[7]. Mammography uptake is lowest among women of Malay ethnicity with only 28.9% reporting having attended mammography within the last 2 years in 2019, compared to their Chinese and Indian counterparts at 40.1% and 41.0% respectively [8]. This ethnic disparity is concerning as it has persisted through the years [8], and it extends also to age-standardized mortality rate of breast cancer. Among those of Malay ethnicity, 19.1 deaths per 100,000 population were reported compared to less than 15.0 in other ethnic groups between 2013 and 2017 [2]. Although ethnic differences in breast cancer mortality cannot be fully explained by differential mammography uptake rate, higher screening uptake remains crucial as those with screen-detected breast cancer had half the risk of mortality of those with clinically-detected breast cancer [9].

Data on breast cancer screening behavior and its determinants specific to the Malay community are lacking. In 2018, a focus group of 27 English-speaking Malay women in Singapore elucidated tradition, beliefs (religious, fatalistic, cultural and intergenerational) such as Islamic values that prohibits revealing of the *aurat* (intimate body parts) and spirituality to influence decision to screen for breast cancer [10]. Beyond this, previous studies have largely focused on the general population. Therefore, specific insights about this community are often underpowered and inconclusive. For instance, Straughan and Seow found fatalism, a belief that some health issues are beyond human control, to be associated with mammography uptake in a survey among predominantly Chinese respondents (84.3%)[11]. However the same authors also reported that Malay women were less fatalistic in a separate study [12] therefore leaving the relationship of fatalism and mammography uptake in this community inconclusive. Other previous investigations also focused on intrapersonal factors like breast cancer knowledge [13] and environmental factors such as screening infrastructure and policies [14]. However, information on interpersonal and community level factors which may influence mammography uptake in the Malay community are lacking.

Recent qualitative studies on the perspectives of health in the Malay community indicate that a widely subscribed vision of health is the notion that health is realized through the equilibrium between the body, the soul and the environment [15]. Unlike the Western framework of health which emphasizes the mind–body dualism in health, the approach to health in the Malay community is more holistic, whereby it extends beyond the individual to include being in a state of balance with the people around them [16]. This approach not only shows the importance of interpersonal and community level factors in influencing health but also highlights that these contextual factors combined with the expectations to uphold Islamic values such as protection of the *aurat* [10] may explain the interethnic disparity in mammography uptake. As Malays residing in Southeast Asia are predominantly Muslim, we reviewed literature elucidated among other Muslim communities globally as well. Other than one study among Muslim women in Jordan that reported interpersonal level relationship with family members, to both encourage breast cancer screening attendance and form competing priorities hindering them from actual attendance [17]; most studies on Muslim women globally reported on community level factors such as positive religious coping, perceived religious discrimination in healthcare [18], modesty concerns [19], and reliance upon traditional or religious healing [20] to hinder screening uptake. These findings highlight potential socio-cultural and religious norms that support and impede breast cancer screening behavior among Muslim women. However, it may be erroneous to extrapolate these findings to the Malay community in Singapore solely on the basis of a shared religion. Unlike most of the aforementioned studies [17–19] where Muslim women were of Africa, Middle-East, Arab and US origins, most Muslim women in Singapore originated from the Malay Peninsula and Indonesia archipelago [9, 21] in Southeast Asia. Therefore, some differences in norms are probable, if not expected.

Disproportionately little attention has been given to Muslims in Southeast Asia – approximately one-fifth of the world’s Muslim population [22]. Similar to Singapore, most studies in this region focused on the general multi-ethnic population. Much of the studies also focused on access to screening facilities and reviewing screening guidelines [23–25] since population-based screening program has yet to be established in these countries [26]. Data on breast cancer screening behavior is thus limited, and among them, most factors elucidated were on intrapersonal level factors such as lack of awareness and knowledge [27–30], and negative perception towards mammography and its outcome [28, 31]. Only one study has reported on interpersonal level factor that is cues from physician was found to facilitate mammogram uptake [31]. No studies thus far have looked into community level factors such as social norms and religious beliefs. To address this knowledge gap, we conducted a sequential mixed-methods needs assessment comprising in-depth interviews followed by a nationwide community-based survey to (i) further explore personal meaning of breast cancer screening attendance and religious interpretation of cancer screening, and then (ii) determine the barriers and facilitators associated with mammography uptake at different levels of the Social-ecological Model (SEM) [32] among Malay-Muslim population in Singapore.

## Methodology

Between August 2017 and May 2018, we conducted semi-structured in-depth interviews (IDI). Findings from this study such as misinformation, perceived religious beliefs, barriers and facilitators guided the design of the questionnaire for our community-based cross-sectional survey which was carried out from October 2018 to February 2020.

### Data collection

In-depth interviews: Singaporean or Singapore Permanent Residents who identify as Malay-Muslim women, aged 40 to 69 years were recruited for this study. We approached 47 eligible women, of whom 49.0% responded with no difference in the response rate between screeners and non-screeners. Maximum variation sampling was used to purposively select 23 participants of diverse ages and educational levels. To ensure representation of women who had and had not attended mammography, we stratified them according to screening status (ever/never screened), age groups (aged 40–49/50–69) and years of schooling (≤ 6 years of schooling, 7–10 years, > 10 years and tertiary education). All interviewers were trained in qualitative research methods and only female researchers (WYS, EQYH, MLW) interviewed the Malay-Muslim women. The interview guide covered (i) general knowledge on breast cancer, (ii) views on whether Muslim women are encouraged to go for breast cancer screening, and (iii) reasons for attending or not attending screening. All interviews were conducted in English although the option for interview to be conducted in Malay language was available. Each interview lasted around 1 to 1.5 hours and were audio-recorded. Participants were given an information sheet explaining the study and S$20 upon completion of the interview in return for their time. Verbal informed consent was obtained before conducting the interviews. The National University of Singapore Institutional Review Board (NUS-IRB) approved this study (reference code H-17–031). Audio-recordings of the interviews were transcribed verbatim and checked for accuracy against the recordings.

Community-based survey: The sample size required to give an estimated prevalence of regular mammography uptake (i.e. every 2 years) of 23.0% [33], with a margin of error of 5.0% at a 95.0% level of confidence is 269. Therefore, after accounting for a response rate of 50.0%, the final computed sample size was 538. As the sampling frame of Malay residents was not available, we used a sampling frame of all households regardless of ethnicity as a proxy. A sampling frame of 850 randomized households stratified by geographical zones and public housing type was obtained from the Singapore Department of Statistics (DOS). Private residences were not included in the sampling frame as 97.5% of Malay residents live in public housing [34] by the Housing Development Board (HDB) and access to private residences are restricted. A letter of invitation was sent to the addresses in the sampling frame minimally 2 weeks before the visit by our interviewer. The letter provided study details including study team contact details and eligibility criteria. This allowed scheduling of an appointment by eligible individuals, informing study team of ineligibility or informing of refusal to participate. Inclusion criteria for this study was female Singapore Citizen or Permanent Resident aged 50–69 years, who identified as being Malay-Muslim. Exclusion criteria were, having a personal history of breast cancer or being unable to provide a coherent response. Unlike our IDI, individuals aged 40–49 years were not included in this phase as the national screening program using mammogram is only offered to this age group if found to be high risk as assessed by their doctor. Interviewers were formally trained by the principal investigator or the study team on interviewing skills, eligibility assessment and replacement strategy to ensure a standardized manner of sampling and administration of questionnaire. The questionnaire was available in both English and Malay language. The questionnaire was translated, and reviewed by at least 3 native Malay speakers on the study team to ensure equivalence of meaning. Verbal consent was taken from participants before conducting the survey. To reduce social desirability bias, the questionnaire was self-administered by participants, with the exception of those who were illiterate or those that reported eyestrain where the questionnaire will be administered by an interviewer fluent in their language of choice (5.0%). Participants were given S$5 and a transport card valued at S$10 upon completion of survey. NUS-IRB approved this study (reference code S-18–305).

### Questionnaire measures

Outcome measures were having undergone mammography at least once, assessed by “Have you ever gone for a mammogram to screen for breast cancer” and regularly go for mammography, assessed by “Have you gone for a mammogram to screen for breast cancer regularly, that is, every 2 years?”. As our IDI highlighted that screening is influenced by factors beyond the intrapersonal level, we based our questionnaire on the SEM, and included constructs that were aligned to the themes found in our IDI. Constructs from the Health Belief Model were included to assess health behavior on the intrapersonal, interpersonal and environmental level, while constructs on religiosity, modesty and fatalism that were identified in studies done among Muslim in other countries were used to assess community level factors. The responses were assessed on 4-point and 5-point scales to facilitate comparison with abovementioned studies. The constructs used are shown below and details on adaptations are included in Additional file 1: Table S1.

1. Health Belief Model (HBM) and extended HBM – HBM postulates that the likelihood of an individual taking on a healthy behavior is dependent on their perception of the HBM constructs – Perceived Susceptibility, Perceived Severity, Perceived Benefit, Perceived Barriers, and Cue to Action [35]. The extended HBM includes additional constructs – Perceived Importance and Consideration of Future Consequences to improve predicting capability of the HBM [36].
2. Psychological Measure of Islam Religiousness (PMIR) Positive Religious Coping (PRC) – The construct describes usage of religious support mechanism to cope, reflecting a secure relationship with Allah [37].
3. PMIR Negative Religious Coping (NRC) – This coping pattern reflects a less secure relationship with Allah, and describes a tenuous and ominous view of the world, marked by religious struggle to find and conserve significance in life [37].
4. PMIR Islamic Religious Internalization-Identification (IRI) – This construct describes adoption of religious beliefs as personal values [37].
5. Modesty – The Modesty construct measures overall perception of modesty and was validated among American Muslims [38].
6. Religious Health Fatalism Questionnaire (RHFQ) – RHFQ measures fatalistic belief – but with a focus on religious belief or the idea of God being in control of health outcomes instead of luck or chance [39].

### Data analysis

In-depth interviews: Transcribed interviews were managed using NVivo Pro Version 12. We explored barriers to and facilitators for mammography, as well as how significant others and referent groups influenced women’s decisions to attend mammography. We also identified factors that characterized women who had undergone mammography (doers) and those who had not (non-doers). The analysis of doers and non-doers enabled identification of culturally appropriate strategies adopted by the doers that could be disseminated to the non-doers. We used a combination of inductive coding and thematic analysis [40] with the two authors (MLW and WYS) independently reading the transcripts to identify codes, patterns of meaning, and themes. Data saturation was reached after interviewing 23 women when no new concepts, with respect to our research questions, could be identified. To organize our findings systematically, we categorized codes into themes on barriers and facilitators. Categorization of themes and subthemes were guided by, but not limited to, the HBM and SEM. Using an inductive data-driven approach [40], we also searched for new themes. Any discrepancies in the codes and themes were resolved by consensus between the two authors, and then by group discussion with the rest of the study team.

Community-based surveys: We conducted data analysis using two software due to availability of software at point of analysis. Preliminary analysis was carried out using R Version 4.0.2 with the psy package and the main statistical tests, using IBM SPSS Statistics for Windows version 26 (IBM Corp., Armonk, N.Y., USA). We first used exploratory factor analysis (EFA) to evaluate factor structure for all theoretical constructs in our questionnaire. Factor solutions identified to be unidimensional were then tested for reliability or internal consistency by computing its Cronbach’s alpha value. Variables belonging to unidimensional factor solution with reasonably high internal consistency (Cronbach alpha > 0.70) were grouped and analyzed as composite score in the main statistical analysis. Other variables were analyzed as single variables with the exception of some variables that were regrouped as a construct and analyzed in the subscale previously validated by the authors (PMIR-NRC and RHFQ) or according to the level of intervention needed to modify the behavior (HBM) (Additional file 1: Table S1).

The main statistical analyses were carried out in two stages: (i) univariable logistic regression to determine variables associated with screening uptake, then (ii) multivariable logistic regression, to produce an adjusted model with multiple independent variables taken into consideration. We conducted a series of univariable logistic regression analyses and only variables with at least moderate strength of statistical evidence of association (Wald’s *p*-value < 0.10) were selected for subsequent multivariable logistic regression model building. We used a forward stepwise approach to build a parsimonious multivariable model. Starting with an empty model, variables with strongest evidence of association were added first and removed accordingly, if addition of subsequent variables caused the variable to no longer be significant at the 0.10 level. Age is the only a priori confounder identified and adjusted in both models as older women, being eligible for a longer period of time, are more likely to have gone for mammogram. We performed the Box-Tidwell test and found that adding a non-linear transformation of age did not improve prediction. Hence, we analyzed age as a continuous variable. We chose the final model based on goodness-of-fit metrics, whereby outputs from the Likelihood Ratio test and Hosmer–Lemeshow test, and Nagelkerke R-square value consistently supported the fit of the final model. We computed Variance Inflation Factor (VIF) for all variables in the final models and confirmed no evidence of multicollinearity (VIF approximately 1) in the final model.

## Results

In-depth Interviews: Of the 23 women interviewed, 10 never had mammography while 13 had mammography at least once (Table 1). We identified 5 themes on facilitators: perceived benefits, cues and support from significant others, symptoms, personal responsibility to care for one’s health and religious belief; and 6 themes on barriers to mammography: perceived low susceptibility, cost, misinformation, religious beliefs, perceived negative outcomes from mammography and distrust of doctors. Selected quotes are found in Additional file 2: Table S2.

**Table 1:** Characteristics of participants for IDI and survey

Demographics	IDI (*n* = 23)	Survey (*n* = 271)
*Age (Mean ± SD)*	–	59.4 (5.5)
*Married*	18 (78.3)	218 (80.4)
*Number of children*	–	
0		21 (7.8)
1		23 (8.6)
2		46 (17.1)
3		93 (34.6)
4 or more		86 (32.0)
*Household income*	–	
< S$2,000		101 (42.6)
S$2,000 – S$3,999		68 (28.7)
S$4,000 – S$5,999		40 (16.9)
≥ S$6,000		28 (11.8)
*Highest education level*		
Primary school	5 (21.7)	89 (33.0)
Secondary school	12 (52.2)	145 (53.7)
Tertiary education^†^	6 (26.1)	36 (13.3)
*Employment status*		
Employed	13 (56.5)	90 (33.2)
*Type of housing*		
HDB 1–2 rooms		30 (11.1)
HDB 3 rooms	12 (52.2) ^§^	68 (25.1)
HDB 4 rooms		119 (43.9)
HDB 5 rooms/	11 (47.8) ^§^	54 (19.9)
*Executive/Others*^‡^		
*Primary care*^¶^	–	235 (87.0)
*Family history of breast cancer*	–	48 (17.8)
*Ever been screened*	13 (56.5)	161 (59.6)
*Regularly screen*^#^	–	73 (31.7)

*HDB* Housing development board. All data provided are number of responses and percentages except where indicated e.g. age. ^†^Tertiary education refers to any post-secondary education including technical education, diploma, and undergraduate. ^‡^Others refer to shop houses or other housing types where participant was recruited from public areas such as food centre during replacement. ^§^Type of housing data collected were HDB ≤ 3 rooms and HDB ≥ 4 rooms. ^¶^Primary care refers to having a doctor they usually see. ^#^Only participants aged above 52 years old who are eligible for regular screening are included in this analysis, *n* = 230

### Facilitators for mammography shared by screeners

1. Perceived benefits – A prominent reason offered by screeners was mammography allows for early detection of breast cancer where it could then be treated effectively.
2. Symptoms – A few participants shared how they had attended screening when they felt abnormalities in their breasts.
3. Cues and support from others – Most screeners shared that they have been reminded or encouraged by their family, friends and doctors to attend mammography. Several participants reported how seeing close ones suffer from breast cancer motivated them to take precautionary measures by attending mammography.
4. Personal responsibility to take care of one’s health – Most screeners explained that it was their personal responsibility to take care of their health. They explained that God (Allah) wanted them to take care of their own health.
5. Religious belief – One participant explained the need to take action and if breast cancer is detected as a result of screening, it was a trial from God (Allah) given to test one’s faith.

### Barriers to mammography shared by screeners and non-screeners

1. Perceived low susceptibility – Some perceived low susceptibility to breast cancer in the absence of a family history in breast cancer or having ever breastfed.
2. Perceived negative outcomes from mammography – Intrapersonal factors ranged from negative perceptions of the mammogram procedure such as pain, to fear of outcome of mammogram such as finding out that they had breast cancer which then requires painful treatment. One screener offered a potential interpersonal level factor which is one’s concern over friends perceiving there was something wrong with them. Some participants offered community-level factors such as modesty concerns, that one’s bodies should only be exposed to their husbands, may also impede uptake.
3. Perceived costs of screening – These included intrapersonal factors such as finance, interpersonal factors such as family responsibilities, and environmental factors such as inconvenience. Some of them did not know that MediSave can be used to pay for mammography for women aged 50 and above.
4. Misinformation on breast cancer and mammogram – Two non-screeners expressed fear of mammogram triggering cancer cells to spread. Some participants did not see a need for mammography because they feel well. They also believed that breast cancer is resultant of breast milk ‘clogging up’ in the breast, that breast cancer cannot be treated even if detected early, and that they were too old for screening and treatments.
5. Religious beliefs (fatalism) – A non-screener reported on the will of God (Allah) in controlling illness whereby some may perceive that if one was destined to have breast cancer, one should accept one’s fate. On that note, some may prefer to depart from this world with a complete body as given by God (Allah).
6. Distrust – Some participants shared on general distrust of doctors having had bad experiences.

**Fig. 1:**
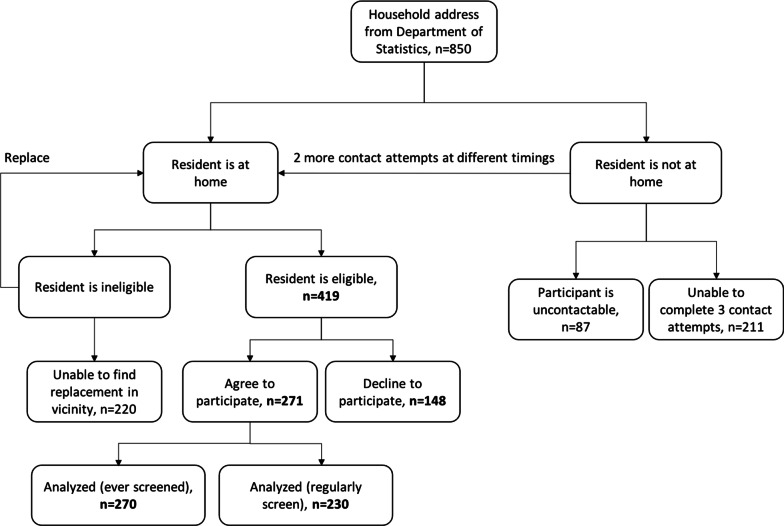
Flow diagram for cross-sectional survey. Ineligible resident will be replaced with next eligible individual within 10 households from the original unit in a descending order. Should no eligible individual be found, interviewer will recruit any eligible individual within the vicinity (100 m radius). Should more than one eligible individual be identified in one household, the KISH grid will be used to randomly select a participant. Of the 717 households, 12.1% were uncontactable after 3 attempts and 29.4% were incomplete contact attempts. A total of 270 surveys were analyzed for ever gone for mammogram due to one missing value of mammogram status and 230 surveys were analyzed for regular mammogram as 41 participants were ineligible for regular mammogram

Community-based Survey: We recruited according to the proportions of female Malay residents in each geographical zones to ensure representativeness of our sample [41]. Of the 419 eligible individuals contacted, 271 responded giving a response rate of 64.7% (Fig. 1). Common reasons for not participating were busy, not feeling well and do not like doing surveys. One was reluctant to participate due to sensitivity of the questions. Of the surveyed individuals, 59.6% reported having gone for a mammogram at least once, 31.0% reported having gone for mammogram within the last 2 years, and 31.7% of those eligible for regular mammogram reported doing so.

Univariable logistic regression analysis (Table 2) identified 25 variables to be associated with ever gone for mammography. These included demographic characteristics; grouped variables namely Interpersonal Barriers, Structural Barriers, PMIR-Punishing Allah Reappraisal (PMIR-PAR) (see Additional file 1: Table S1 for details on subscale); and single items from the Perceived Benefit, Perceived Susceptibility, Perceived Importance, Cues to Action, PMIR-IRI, Modesty, and some themes from our IDI. We also identified 20 variables for regular mammography uptake including demographic characteristics; grouped variables namely Interpersonal Barriers, PMIR-PAR, Divine Provision, and Structural Barriers; and single items from Perceived Benefit, Perceived Susceptibility, Consideration of Future Consequences, Perceived Importance, Cues to Action constructs and some themes from our IDI.

**Table 2:** Unadjusted odds ratio (OR) for variables considered in multivariable logistic regression

Sociodemographic Characteristic	Ever gone (*n* = 270)^†^		Regular (*n* = 230)^‡^	
	OR (95%CI)	*P*-value	OR (95%CI)	*P*-value
*Age* (*n*_e_ = 267, *n*_r_ = 230)	1.04 (1.00, 1.09)	< 0.10	0.99 (0.93, 1.05)	0.62
*Household Income* (*n*_e_ = 236, *n*_r_ = 199)				
< S$2,000	Ref	0.45	Ref	< 0.01
S$2,000–S$3,999	1.24 (0.67, 2.32)		1.19 (0.55, 2.56)	
S$4,000–S$5,999	1.36 (0.64, 2.89)		2.89 (1.19, 7.02)	
≥ S$6,000	2.05 (0.82, 5.08)		4.33 (1.66, 11.29)	
*Highest Education Level* (*n*_e_ = 269, n_r_ = 229)				
Primary Education	Ref	0.15	Ref	< 0.05
Secondary Education	1.38 (0.81, 2.35)		2.00 (1.05, 3.79)	
Tertiary Education	2.23 (0.96, 5.19)		4.00 (1.52, 10.55)	
*Type of Housing* (*n*_e_ = 270, *n*_r_ = 230)				
HDB 1–2 Rooms	Ref	0.38	Ref	< 0.10
HDB 3 Rooms	0.69 (0.29, 1.65)		3.18 (0.97, 10.43)	
HDB 4 Rooms	1.10 (0.48, 2.49)		1.97 (0.62, 6.26)	
HDB 5 Rooms/Others	1.23 (0.49, 3.08)		3.84 (1.13, 13.02)	
*Personal Doctor* (*n*_e_ = 269, *n*_r_ = 230)				
Yes	2.86 (1.37, 5.96)	< 0.01	4.21 (1.23, 14.47)	< 0.05
*Family History* (*n*_e_ = 269, *n*_r_ = 230)				
Yes	1.80 (0.91, 3.54)	< 0.10	3.13 (1.57, 6.26)	< 0.01
*Attended a prayer in the mosque in the last 1 year* (*n*_e_ = 270, *n*_r_ = 230)				
Yes	1.94 (1.13, 3.32)	< 0.05	1.50 (0.79, 2.83)	0.22
**Grouped Variables**				
**Interpersonal Level:**				
*HBM Perceived Barriers, Interpersonal *(*n*_e_ = 248, *n*_r_ = 210)				
Low	Ref	< 0.01	Ref	< 0.10
Moderate	0.45 (0.25, 0.83)		0.51 (0.26, 1.00)	
High	0.34 (0.17, 0.67)		0.48 (0.22, 1.04)	
**Community Level:**				
*PMIR Punishing Allah Reappraisal* (*n*_e_ = 255, *n*_r_ = 216)				
Low	Ref	< 0.05	Ref	< 0.10
Moderate	0.45 (0.24, 0.83)		0.73 (0.38, 1.43)	
High	0.51 (0.27, 0.98)		0.41 (0.19, 0.86)	
*RHFQ Divine Provision* (*n*_e_ = 259, *n*_r_ = 222)				
Low	Ref	0.15	Ref	< 0.01
Moderate	0.57 (0.31, 1.04)		0.36 (0.18, 0.74)	
High	0.90 (0.48, 1.66)		0.38 (0.19, 0.77)	
**Environmental Level:**				
*HBM Perceived Barriers, Structural* (*n*_e_ = 266, *n*_r_ = 226)				
Low	Ref	< 0.01	Ref	< 0.01
Moderate	0.46 (0.25, 0.83)		0.38 (0.19, 0.75)	
High	0.40 (0.21, 0.74)		0.20 (0.09, 0.45)	
**Variables as Single Items**				
**Intrapersonal Level:**				
*HBM Perceived Benefit: Breast cancer if detected early can be cured* (*n*_e_ = 268, *n*_r_ = 230)				
Agree	Ref	< 0.01	Ref	0.64
Neutral	0.23 (0.08, 0.61)		0.59 (0.19, 1.85)	
Disagree	0.11 (0.03, 0.41)		0.82 (0.25, 2.71)	
*HBM Perceived Benefit: If breast cancer is detected early, chances of cure are very high* (*n*_e_ = 269, *n*_r_ = 230)				
Agree	Ref	< 0.01	Ref	0.40
Neutral	0.24 (0.07, 0.82)		0.44 (0.09, 2.12)	
Disagree	0.15 (0.05, 0.46)		0.57 (0.18, 1.80)	
*HBM Perceived Benefit: There are medical tests now that can detect breast cancer in its very early stage* (*n*_e_ = 267, *n*_r_ = 228)				
Agree	Ref	< 0.01	Ref	0.61
Neutral	0.33 (0.16, 0.67)		0.64 (0.26, 1.57)	
Disagree	0.36 (0.10, 1.31)		0.86 (0.21, 3.43)	
*HBM Perceived Benefit: Cancer is like a death sentence; if you get it, you will surely die from it* (*n*_e_ = 266, *n*_r_ = 228)				
Disagree	Ref	0.77	Ref	< 0.10
Neutral	0.79 (0.42, 1.49)		0.99 (0.49, 2.02)	
Agree	0.95 (0.52, 1.73)		0.45 (0.22, 0.93)	
*HBM Perceived Susceptibility: I worry about getting breast cancer all the time* (*n*_e_ = 267, *n*_r_ = 229)				
Disagree	Ref	0.29	Ref	< 0.05
Neutral	1.32 (0.69, 2.52)		1.97 (0.94, 4.12)	
Agree	1.59 (0.90, 2.81)		0.74 (0.38, 1.43)	
*HBM Perceived Susceptibility: I can prevent myself from getting breast cancer* (*n*_e_ = 265, *n*_r_ = 228)				
Agree	Ref	< 0.05	Ref	0.87
Neutral	0.49 (0.28, 0.86)		1.18 (0.61, 2.27)	
Disagree	0.47 (0.21, 1.04)		1.15 (0.48, 2.75)	
*EHBM Consideration of Future Consequences: I only act to satisfy my immediate concerns, the future will take care of itself *(*n*_e_ = 252, *n*_r_ = 217)				
Disagree	Ref	0.13	Ref	< 0.05
Neutral	0.46 (0.22, 0.98)		0.81 (0.36, 1.82)	
Agree	0.64 (0.32, 1.26)		0.43 (0.21, 0.89)	
*EHBM Perceived Importance: It is important for me to go for a mammogram to screen for breast cancer* (*n*_e_ = 255, *n*_r_ = 218)				
Agree	Ref	< 0.01	Ref	< 0.01
Neutral	0.16 (0.07, 0.36)		0.15 (0.03, 0.65)	
Disagree	0.06 (0.01, 0.25)		0.12 (0.02, 0.94)	
*IDI: All mammogram screening are done by women* (*n*_e_ = 266, *n*_r_ = 227)				
Agree	Ref	< 0.05	Ref	0.14
Neutral	0.30 (0.14, 0.66)		0.45 (0.15, 1.39)	
Disagree	0.49 (0.18, 1.31)		0.32 (0.07, 1.46)	
*IDI: I am too old to go for mammogram* (*n*_e_ = 268, *n*_r_ = 229)				
Disagree	Ref	0.17	Ref	< 0.01
Neutral	0.63 (0.31, 1.27)		0.28 (0.10, 0.76)	
Agree	0.59 (0.31, 1.12)		0.19 (0.07, 0.50)	
*IDI: I must have symptoms first before I decide to go for mammogram* (*n*_e_ = 267, *n*_r_ = 229)				
Disagree	Ref	< 0.01	Ref	< 0.01
Neutral	0.29 (0.13, 0.62)		0.39 (0.16, 0.98)	
Agree	0.30 (0.17, 0.53)		0.17 (0.08, 0.38)	
**Interpersonal Level**				
*HBM Cues to Action: My friends have told me to go for mammogram* (*n*_e_ = 266, *n*_r_ = 228)				
Agree	Ref	< 0.10	Ref	0.58
Neutral	0.47 (0.24, 0.93)		0.63 (0.27, 1.51)	
Disagree	1.18 (0.63, 2.24)		0.87 (0.44, 1.76)	
*HBM Cues to Action: I would like to be accompanied by my family or friend to go for mammogram* (*n*_e_ = 267, *n*_r_ = 229)				
Agree	Ref	< 0.01	Ref	< 0.10
Neutral	0.94 (0.47, 1.88)		1.32 (0.56, 3.12)	
Disagree	3.27 (1.81, 5.89)		2.04 (1.11, 3.76)	
*HBM Cues to Action: My personal doctor had recommended mammogram to me* (*n*_e_ = 269, *n*_r_ = 230)				
Agree	3.20 (1.92, 5.35)	< 0.01	3.03 (1.58, 5.80)	< 0.01
*HBM Cues to Action: My nurse or other health care provider (other than the doctor) had recommended mammogram to me* (*n*_e_ = 269, *n*_r_ = 230)				
Agree	2.85 (1.72, 4.72)	< 0.01	2.04 (1.14, 3.66)	< 0.05
**Community Level**				
*PMIR Islamic Religious Internalization-identification: I pray because I find it satisfying* (*n*_e_ = 269, *n*_r_ = 230)				
Agree	0.14 (0.02, 1.10)	< 0.10	0.68 (0.19, 2.49)	0.56
*Modesty: When I am in a mixed gender gathering or outside of the home, I cover my entire body except my hands and face* (*n*_e_ = 266, *n*_r_ = 226)				
Agree	Ref	< 0.05	Ref	0.22
Neutral	1.95 (0.83, 4.56)		1.26 (0.54, 2.96)	
Disagree	0.52 (0.25, 1.07)		0.44 (0.16, 1.21)	
*Modesty: I have delayed seeking medical care when no woman doctor is available to see me* (*n*_e_ = 267, *n*_r_ = 228)				
Agree	Ref	< 0.10	Ref	0.27
Neutral	0.43 (0.20, 0.93)		1.77 (0.74, 4.23)	
Disagree	0.63 (0.33, 1.19)		0.99 (0.49, 2.01)	
*IDI: The breast should be ‘intact’ in my body so that I am complete when I leave this world* (*n*_e_ = 268, *n*_r_ = 230)				
Disagree	Ref	< 0.10	Ref	0.25
Neutral	0.47 (0.24, 0.91)		0.65 (0.30, 1.40)	
Agree	0.89 (0.50, 1.58)		0.60 (0.32, 1.12)	
*IDI: I prefer to go to my masseuse or traditional healer to screen for breast cancer *(*n*_e_ = 268, *n*_r_ = 230)				
Agree	Ref	< 0.01	Ref	< 0.10
Neutral	0.33 (0.16, 0.69)		0.48 (0.19, 1.23)	
Disagree	0.30 (0.12, 0.81)		0.24 (0.05, 1.10)	

*Ref* Reference group. ^†^One missing value. ^‡^41 missing values as only participants aged above 52 years old who are eligible for regular screening are included in this analysis. *n*_e_, sample size for ever gone. *n*_r_, sample size for regular mammogram. This table includes only variables with *P*-value < 0.10. Only these variables were considered in the final multivariable logistic regression analysis. *HDB* Housing Development Board. *HBM* Health Belief Model. *PMIR* Psychological Measure of Islam Religiousness. *RHFQ* Religious Health Fatalism Questionnaire. EHBM, Extended HBM. IDI, In-depth Interviews. HBM Perceived Barriers, Interpersonal is a composite score for 4 variables: i) I am afraid that I will be judged by my community if I go for mammogram, ii) It is difficult to find someone to take over my responsibilities at home if I have to go for mammogram, iii) It is difficult to find someone to accompany me for mammogram, and iv) I must ask my husband for permission before I go for mammogram. HBM Perceived Barriers, Structural is a composite score for 3 variables: i) Mammogram is expensive, ii) It is inconvenient for me to go to the clinic or hospital for mammogram, and iii) It is difficult for me to take time to go to the clinic or hospital for mammogram

Table 3 shows results from the final multivariable logistic regression models, testing the relationships of ever gone for mammography and regular mammography uptake by the aforementioned variables. We found 7 variables to be significantly associated with ever gone for mammography and 5 variables with regular mammography uptake. Age was found to be associated with ever gone for mammography even after accounting for the other 6 variables, where for every additional year, there is an increase in odds of mammography by 7% (adjusted odds ratio, aOR = 1.07, 95% CI: 1.01, 1.14). The model also showed those that received cues to attend mammography from nurses had 4.12 times (aOR = 4.12, 95% CI: 2.04, 8.33) the odds of going for mammography at least once compared to those that did not receive such cues, regardless of their other beliefs and age. In terms of beliefs, we found at least 67% lesser odds of attending mammography at least once among those that perceived they must have symptoms before going for mammography (aOR = 0.33, 95% CI: 0.16, 0.70), those that more strongly appraise difficulties in life as punishment from Allah (PMIR-PAR) (aOR = 0.32, 95%CI: 0.13, 0.79), those that did not perceive mammography to be important (aOR = 0.12, 95%CI: 0.02, 0.66) and those that did not perceive breast cancer is curable if detected early (aOR = 0.05, 95%CI: 0.01, 0.30), compared to the converse of those beliefs. We also found those that held a neutral perception towards dressing modesty had 3.07 times the odds of attending mammography at least once (aOR = 3.07, 95%CI: 0.87, 10.83) compared to those that agreed to the need for modesty in appearance. The prevalence of the religious beliefs, PMIR-PAR and modesty, were relatively high, with 29.4% scoring higher in PMIR-PAR and 74.4% agreeing to the need for modesty in appearance.

**Table 3:** Adjusted odds ratio of ever gone for mammogram and regular mammogram uptake

Variable	Ever Gone (*n* = 270)^†^		Regular (*n* = 230)^‡^	
	aOR (95%CI)	*p*-value	aOR (95%CI)	*p*-value
*Age* (*n*_e_ = 267, *n*_r_ = 230)	1.07 (1.01, 1.14)	< 0.05	1.07 (0.98, 1.17)	0.15
*Household Income* (*n*_e_ = 236, *n*_r_ = 199)				
< S$2,000	–	–	Ref	< 0.05
S$2,000–S$3,999			1.04 (0.43, 2.39)	
S$4,000–S$5,999			3.06 (1.07, 8.79)	
≥ S$6,000			3.76 (1.11, 12.72)	
*PMIR Punishing Allah Reappraisal *(*n*_e_ = 255, *n*_r_ = 216)				
Low	Ref	< 0.05	–	–
Moderate	0.29 (0.12, 0.68)			
High	0.32 (0.13, 0.79)			
*HBM Perceived Barriers, Structural *(*n*_e_ = 266, *n*_r_ = 226)				
Low	–	–	Ref	< 0.10
Moderate			0.41 (0.17, 0.97)	
High			0.40 (0.14, 1.14)	
*EHBM Perceived Importance: It is important for me to go for a mammogram to screen for breast cancer* (*n*_e_ = 255, *n*_r_ = 218)				
Agree	Ref	< 0.01	–	–
Neutral	0.17 (0.07, 0.46)			
Disagree	0.12 (0.02, 0.66)			
*HBM Cues to Action: My nurse or other health care provider had recommended mammogram to me* (*n*_e_ = 269, *n*_r_ = 230)				
Agree^§^	4.12 (2.04, 8.33)	< 0.01	–	–
*HBM Cues to Action: My personal doctor had recommended mammogram to me* (*n*_e_ = 269, *n*_r_ = 230)				
Agree^§^	–	–	3.45 (1.55, 7.66)	< 0.01
*HBM Perceived Benefit: Breast cancer if detected early can be cured* (*n*_e_ = 268, *n*_r_ = 230)				
Agree	Ref	< 0.01	–	–
Neutral	0.26 (0.08, 0.88)			
Disagree	0.05 (0.01, 0.30)			
*HBM Perceived Susceptibility: I worry about getting breast cancer all the time* (*n*_e_ = 267, *n*_r_ = 229)				
Disagree	–	–	Ref	< 0.05
Neutral			3.19 (1.21, 8.43)	
Agree			0.90 (0.38, 2.09)	
*IDI: I must have symptoms first before I decide to go for mammogram* (*n*_e_ = 267, *n*_r_ = 229)				
Disagree	Ref	< 0.01	Ref	< 0.01
Neutral	0.29 (0.10, 0.84)		0.50 (0.15, 1.67)	
Agree	0.33 (0.16, 0.70)		0.19 (0.07, 0.51)	
*Modesty: When I am in a mixed gender gathering or outside of the home, I cover my entire body, except my hands and face* (*n*_e_ = 266, *n*_r_ = 226)				
Agree	Ref	< 0.10	–	–
Neutral	3.07 (0.87, 10.83)			
Disagree	0.50 (0.20, 1.28)			

*Ref,* Reference group.—Variables not found to be significantly associated with mammogram uptake in final model. ^†^One missing value. ^‡^41 missing values as only participants aged above 52 years old who are eligible for regular screening are included in this analysis. *n*_e_, sample size for ever gone for mammogram. *n*_r_, sample size for regular mammogram. ^§^Binary data, Agree vs Disagree. *PMIR* Psychological Measure of Islam Religiousness. *HBM* Health Belief Model. *EHBM* Extended HBM. *IDI* In-depth interviews. *HBM* Perceived Barriers, Structural is a composite score for 3 variables: i) Mammogram is expensive, ii) It is inconvenient for me to go to the clinic or hospital for mammogram, and iii) It is difficult for me to take time to go to the clinic or hospital for mammogram

We did not find age to be significantly associated with regular mammography uptake. Individuals with household income of S$4,000 and above had about 3 times (aOR = 3.06, 95%CI: 1.07, 8.79; aOR = 3.76, 95%CI: 1.11, 12.72) the odds of attending regular mammography compared to those reporting a household income of < S$2,000. Similarly, those that received cues from their doctor for mammogram uptake had 3.45 times (aOR = 3.45, 95%CI: 1.55, 7.66) the odds of attending mammogram regularly compared to those that did not receive such cues. In terms of beliefs, we found at least 60% lesser odds of attending mammogram regularly among those that hold a stronger perception of structural barriers to screening (aOR = 0.40, 95%CI: 0.14, 1.14), and those that perceived the need for symptoms before going for a mammogram (aOR = 0.19, 95%CI: 0.07, 0.51) compared to those that perceived otherwise. We also found those that were neutral about their susceptibility towards breast cancer had 3.19 (aOR = 3.19, 95%CI: 1.21, 8.43) times the odds of attending regular screening compared to those that did not perceive themselves to be susceptible towards breast cancer.

## Discussion

### Key findings

Our sequential mixed-methods study aimed to understand personal and religious meaning of breast cancer screening, and to identify barriers to and facilitators for mammogram uptake among Malay-Muslim women in the community. The IDI highlighted that decisions on mammogram uptake among the Malay-Muslim community in Singapore did not only depend on intrapersonal factors, but also their interpersonal relationships, community level factors such as their normative religious beliefs, and environmental factors such as screening facilities’ availability and access. Our nationwide survey results support the IDI findings, whereby mammography uptake was associated with multilevel factors. Factors that were associated with ever gone for mammogram were age, perceived benefits of saving lives from early detection, perceived importance of mammogram, cues from health care professionals and modesty concerns, appraisal of difficulties in life as a punishment from Allah (PMIR-PAR), and needing symptoms before deciding to go for mammogram. Factors associated with regular mammography uptake included household income, perceived structural barriers to screening, needing symptoms before deciding to go for mammogram, cues from health care professionals, and perceived susceptibility to breast cancer.

### Interpretation of findings

To our knowledge this is the first study locally, to assess and compare factors associated with both ever gone for mammography, and regular mammography uptake. Most studies viewed mammography uptake as a single behavior and therefore assessed either uptake [12–14] or the intention to screen [42]. Of note, we found first and regular mammography uptake were only similar on two accounts in that, receiving cues from healthcare professionals facilitated both uptakes, and perception of needing symptoms prior to mammogram hindered both uptakes. Beyond this, ever gone for mammography and regular mammography uptake were facilitated and hindered differently. While ever gone for mammography was associated with perceptions of mammography and more deep-seated values such as PMIR-PAR and modesty concern, regular mammography was associated with household income, perceived structural barriers such as cost and ease of access, and one’s perceived susceptibility. One possible explanation for the differences in associations is that, positive perceptions on mammogram benefits and importance may encourage first uptake, but is insufficient to sustain regular uptake possibly due to a null result which may in turn reinforce belief of low susceptibility. On the other hand, deep-seated values may impede first uptake, however having overcome these initial barriers, affordability and ease of access matters more in decision for subsequent uptakes.

Such deep-seated values may have contributed to the observed interethnic disparity in mammography uptake too. Appraisal of difficulties in life as punishment from Allah (PMIR-PAR), and modesty concern relating to appearance have not been reported to be associated with mammography uptake or other cancer screening among other ethnic groups in Singapore – suggesting that these factors are likely to be unique to this community. Concerns on removal of clothing for medical tests [11] and preference for healthcare professional of certain gender [14] have been reported previously as barriers to screening among Asians in general. Both these factors were assessed in our survey but were outcompeted in our final multivariable logistic model by modesty concern relating to one’s dressing or appearance. Individuals holding a neutral perception towards modesty concerns in appearance, which is a core value of the Islamic faith [43], were most likely to attend screening compared to those that agreed to it. As the Islamic faith prohibits revealing of the *aurat* [10] with the exception of an emergency such as illnesses [44], our findings show that those that perceived this exception to include preventative health measures such as cancer screening were more likely to go for mammography. More importantly, our findings showed that those that place higher value on upholding modesty in their appearance were less likely to have ever gone for mammography screening, demonstrating that this religious priority does take precedence over mammography screening among many in this community. Prioritization of modesty in appearance over health is not limited to mammography screening and has been put forth to hinder Singaporean Malay women from taking part in physical activity in public spaces [15], despite physical activity being explicitly encouraged in the Islamic faith [45]. Compared to physical activity, cancer screening has not been directly addressed in religious guidance [44], potentially making the decision for cancer screening over preservation of modesty even harder, to the extent that breast cancer screening has been reported to be viewed as a taboo [10].

PMIR-PAR, or religious coping in general, has not been discussed in local or regional cancer screening literature. Hence, our finding which suggests, resigning to punishments given by Allah to lead to a deferment of mammography uptake among Muslims in Singapore, is a novel finding in this region. Notably, no association was found between PMIR-PAR and mammography uptake among Muslims in the US. Instead, positive religious coping (PMIR-PRC) was negatively associated with mammography uptake among Muslims in the US [18]. Taken together, our findings demonstrate strong association of religious coping with deferment of mammography uptake among Muslims, although the specific religious coping style differed with the regions. The reason for this difference in religious coping styles is unclear, but may be partly explained by differences in educational level and cultural background of women in the two studies. Of the respondents in the US, 65.0% were of tertiary education, compared to 13.3% in our study. Also, the study participants in the US comprised Muslim women of Arab, African and South Asian ethnicity with one third having only lived in US for the past 20 years, while our study comprised women of Malay ethnicity which are the original inhabitants of Singapore. Further qualitative inquiry is needed to understand the influence of religious coping on breast cancer screening, and the role of culture in this relationship. While household income and perceived structural barriers to screening are unlikely to be factors unique to this community, these factors may increase the interethnic disparity in mammography uptake, particularly regular uptake. This was confirmed by our study, which showed an independent significant association of household income with regular uptake. Additionally, those of Malay ethnicity report the lowest average household income in Singapore [46]. Given the availability of subsidies and facilities across Singapore, policy makers and health care providers may attribute perceived structural barriers to screening in this community, such as cost, inconvenience and lack of time, to a lack of knowledge on access and hence advocate for more public education on available subsidies and facilities. However, this action is unlikely to fully resolve the perceived barriers in this community. We found perceived structural barriers to be associated with regular screening but not with ever screening, suggesting that these women have acquired the knowledge, having gone for mammography for the first time. There could be other explanations such as Malay women prioritizing the needs of their loved ones over their health hence leading to them not attending screening regularly. This has been reported to hinder women from attending breast cancer screening in Jordan [17], and has been discussed elaborately in a series of in-depth interviews on heart health, whereby Singaporean Malay women described management of their heart health as “looking after their loved ones’ needs, no matter the strife” [47]. Given that these were qualitative studies, further investigation is needed to establish the relationship of prioritization the needs of loved ones with their health screening behaviour.

### Study limitations and strengths

Our study had the following limitations. Despite efforts to maintain representativeness and reduce non-contactable individuals for the survey, number of households that remained uncontactable (12.1%) or have not completed contact attempts were relatively high (29.4%) (Fig. 1). We were not able to collect demographic variables from non-responders since verbal consent was not given, and therefore are not able to ascertain if non-responders significantly differed from responders. Also, the religious constructs were not validated in this region prior to data collection. However, these constructs were validated by the original authors, and were assessed as individual variables when found to be not unidimensional in EFA and scored low on inter-item reliability score. Finally, this research was conducted before the COVID-19 pandemic, hence potential barriers such as fear of attending health care facilities [48] due to COVID were not assessed. This might have changed some barriers and facilitators elucidated, but not the community-level norms relating to more deep-seated religious values.

Despite these limitations, our study has many strengths. First, our study assessed and demonstrated that different factors influenced the two behavioral outcomes, hence showing the need for different interventions and public health communication messages in targeting these behaviors. Second, using a qualitative inquiry before the quantitative survey ensured that we gained insights into all possible reasons for and against mammogram uptake before systematically determining factors that were associated with mammogram uptake in a representative sample in this community. Not only did the qualitative study prompt us to combine the Social Ecological Model with the individual-level Health Belief Model to assess multi-level factors associated with screening in the survey, it also helped explain prevalent misinformation. This is crucial in designing health communication messages specific for this community. Third, our findings on the influence of religion on mammography uptake that are potentially unique to the Malay-Muslim community helped to explain the persistent lower mammography uptake in this community compared to other ethnic groups despite equal access to health care services.

### Public health and research implications

Our finding on cues from healthcare professionals to predict mammography uptake highlights the crucial role healthcare professionals play in encouraging screening in this community. Our finding on perception of needing symptoms prior to screening on the other hand, indicates the need for health education to specifically clarify that mammography can detect breast cancer prior to development of physical symptoms. Given the strong association observed between PMIR-PAR and mammography uptake and the high prevalence of such beliefs (29.4%), clearly, breast cancer screening interventions and its accompanying health messages should be jointly developed with religious leaders and the Islamic Religious Council of Singapore. We need to reduce perceptions on punishing appraisals, and clarify religious teachings on health preventive measures so as to reduce mental conflicts, particularly for those who highly value modesty in appearance (74.4%), and facilitate decision-making on cancer screening. We also need to encourage conversations among Malay women and among their loved ones, to emphasize prioritization of one’s own health and therefore foster a shift in norms with regards to women’s role in this community. In a multi-ethnic population in Singapore, ethnic-specific screening interventions should be part of the strategy to reach out to minority ethnic groups so as to increase equity in mammography uptake. Notably, our findings will have public health implications for our neighboring countries such as Malaysia and Indonesia, which not only share cultural roots with Malay-Muslims in Singapore, but also make up a larger proportion of their population. Mammogram uptake in Malaysia remains low, with approximately 25.0% having ever gone for mammogram in the general population [49]. In Indonesia, breast self-examination and clinical breast examination are currently still the recommended screening test, with one study reporting only 0.1% of its participants ever going for mammogram [50]. To promote mammography in these countries, it may be useful to conduct behavioral research on breast cancer screening that incorporates religious and cultural factors. This too applies to behavioral research on other cancer screening programs in Singapore. Finally, given our findings on the differences in factors related to ever gone for mammography and regular mammography, future behavioral research should assess these behaviors separately to facilitate development of differentiated strategies relevant to the differing needs of the two distinct (ever and regular) screener groups.

In summary, our study added significantly to the literature on mammography uptake behaviors among the Malay-Muslim community in Singapore. We identified factors that could have led to the ethnic disparity in mammogram uptake and also proposed actionable strategies to address this disparity. Notably, our study highlighted multiple key players that should be involved in future mammography promotion. As the Malay-Muslim community in Singapore shares its roots with Malaysia and Indonesia, community-level factors elucidated can be applied to the Muslim community there too.

## Supplementary Information

**Additional file 1: Table S1. **Constructs used in survey, adaptation done to original construct.

**Additional file 2: Table S2. **Quotes accompanying themes elucidated from IDI.

## Data Availability

The data (transcripts from individual in-depth interviews and survey) that support the findings of this study are available on request from the corresponding author. The data are not publicly available due to privacy or ethical restrictions.
