# Supplementary material for: Multi-level determinants of breast cancer screening among Malay-Muslim women in Singapore: a sequential mixed-methods study
Source: BMC Womens Health. 2022 Sep 19;22:383. doi: 10.1186/s12905-022-01972-y (PMC9483897; doi:10.1186/s12905-022-01972-y)
Supplement: Supplementary file 1 — Additional file 1: Table S1. Constructs used in survey, adaptation done to original construct. [file 12905_2022_1972_MOESM1_ESM.docx]

| Supplementary Table 1. Constructs used in survey, adaptation done to original construct | | |
| --- | --- | --- |
| **Constructs** | **Adaptation for Survey** | **Details on Regrouping of Variables before Analysis** |
| Health Belief Model (HBM) | We included the constructs Perceived Susceptibility, Perceived Severity, Perceived Benefit, Perceived Barriers, and Cue to Action from the HBM [35]. The construct Self-efficacy was not included in this questionnaire since mammography procedure is not performed by the individual themselves and therefore no ability nor confidence is required. We also included two of the four variables from the extended HBM [36] that were relevant to screening behavior, Perceived Importance and Consideration of Future Consequences. | Variables under this construct were further grouped according to the level of intervention needed to modify behavior before analysis. We define i) Intrapersonal Barriers as personal or psychological barriers to screening, ii) Interpersonal Barriers as interpersonal influences impeding screening, and iii) Structural Barriers as perception of infrastructure, environment or policies impeding screening. |
| Psychological Measure of Islam Religiousness (PMIR) Positive Religious Coping (PRC) | - | - |
| PMIR Negative Religious Coping (PMIR-NRC) | - | Three of five variables under this construct were grouped and analyzed as Punishing Allah Reappraisal (PMIR-PAR), a subscale that was identified by the original author, describing interpretation of difficulties in life as a punishment from Allah [37]. |
| PMIR Islamic Religious Internalization-Identification (PMIR-IRI) | We only included two of the original five variables to avoid repetition as these variables were similar to one another. | - |
| Modesty | We excluded three questions and adapted one question to reduce sensitivity of the questions and increase its relevance. | - |
| Religious Health Fatalism Questionnaire (RHFQ) | We used ‘Allah’ instead of ‘God’ to increase relevance to our Muslim respondents [39]. | We analyzed our data according to the 2 subscales, Divine Provision and Destined Plan, identified by the original author [39]. |
| - No adaptation or regrouping was carried out for the construct. | | |
