# Supplementary material for: Multi-level determinants of breast cancer screening among Malay-Muslim women in Singapore: a sequential mixed-methods study
Source: BMC Womens Health. 2022 Sep 19;22:383. doi: 10.1186/s12905-022-01972-y (PMC9483897; doi:10.1186/s12905-022-01972-y)
Supplement: Supplementary file 2 — Additional file 2: Table S2. Quotes accompanying themes elucidated from IDI. [file 12905_2022_1972_MOESM2_ESM.docx]

| Supplementary Table 2. Quotes accompanying themes elucidated from IDI. | |
| --- | --- |
| **Themes** | **Quotes** |
| Facilitators for mammography  Perceived benefit – Mammography was perceived to enable early detection therefore allowing effective treatment and their lives and breast to be saved. Another participant elaborated on how this will then allow her to spend more time with her loved ones. | “[…] You can know earlier. Better, the better lah, earlier, earlier. Can know earlier, can take medicine earlier right.”  M32, screener with primary education, ≥ 50 years  “Ahh… mammogram, it’s- it’s good if you can, because from the mammogram, you can detect the early signs of cancer, that is the important thing. […] So, if, if the detection is early yeah, it’s better for you, you can save your life, you can save your breast. Right?”  M15, screener with tertiary education, 40-49 years  “[…] Then I must (be) health conscious. I got so many children, I got so many grandchildren ma. Like [laughs], want to see them everyday […]”  M16, screener with secondary education, ≥ 50 years |
| Symptoms – Some shared how they had attended screening when they felt abnormalities in their breasts. They reported how the experience made them more vigilant about going for regular mammography. | “[…] Suddenly I… what make me go, is, I had a pain here [pats her breast].”  M21, screener with secondary education, ≥50 years  “[…] So from the biopsy one ah, the.. ah.. not cancerous. It’s only calcium […] But then it’s erm.. you have to.. er.. go your- go for our medical check-up (regular mammogram) lah. It’s good.”  M15, screener with tertiary education, 40-49 years |
| Cues and support from others – Most of the screeners shared that they have been reminded or encouraged by their husbands, siblings and children, friends and doctors to attend mammography. Three regular screeners reported that their husbands encouraged them to do so. One participant recounted how her husband had made her appointment and accompanied her to the hospital. Several participants reported how seeing family members or friends suffer from breast cancer motivated them to take precautionary measures by attending mammography. They described how those afflicted with late-stage cancer were unable to carry out their daily activities, passed away at a young age, and one even had her husband leaving her when she had both breasts removed as a result of late cancer detection from not attending screening. | “ […] my husband always remind me: “You must go, because you quite old”.”  M14, screener with secondary education, ≥50 years  “Arrangement ah… I call. Normally my husband call (to make appointments). […]”  M32, screener with primary education, ≥ 50 years  “Aiyah [expression of disappointment] sad lah, very sad. Scared also […] That one breast cancer, that one also spread the other hand. The hand cannot, cannot.. cannot lah, cannot do anything lah […]”  M32, screener with primary education, ≥ 50 years  “[…] Then they (the breast cancer) like stage 4 already right, then no choice, have to cut or throw right. […] [crying] But luckily, she is independent lah. After divorce, she with her children lah.”  M49, non-screener with primary education, 40-49 years |
| Personal responsibility to take care of one’s health – Most screeners, regardless of their educational level, explained that it was their personal responsibility to take care of their health by attending mammography. Also, they explained that God (Allah) wanted them to take care of their own health. Others explained how no one except themselves could help them to be healthy. | “[…] we are supposed to be proactive, so not just sit back and say “Oh, this is the will of God, I am supposed to die this way.”[…] in our religion, we say that every sickness, there is a cure. So go and find the cure.”  M50, Screener with tertiary education, ≥50 years  “I only decide for my healthy (health), only that. Because I, I got children, I got husband, I got family. If I not well, who wants to look after, who wants to look after me? [laughs] It is ourselves right?”  M32, screener with primary education, ≥50 years |
| Religious belief – A screener explained the need to take action and if breast cancer is detected as a result of screening, it was a trial from God (Allah) given to test one’s faith. Hence, it should be viewed as a challenge to overcome. | “[…] I might get also, anytime, so how do I, how’s my approach? So everything is a test ah. God’s test. Yah, it’s how you solve the problem. It’s either you go nearer to him or you go far away from him. That’s the choice. […]”  M50, screener with tertiary education, ≥50 years |
| Barriers to mammography  Perceived low susceptibility – Two non-screeners, one with tertiary education and another with 6 years of education, did not perceive themselves at risk of breast cancer because they did not have a family history. Another participant shared that prior discovering a symptom, she had perceived herself to be of low susceptibility since she has breastfed. | “[…] So, I thought like maybe partly it could be genetic, genetic. So erm, not sure, let’s say we don’t really have a serious history in the family side, actually that is the reason why I feel like I.. don’t have the need to check for (breast cancer) now.”  M48, non-screener with tertiary education, 40-49 years.  “ […] my mindset always think, ah… because I breastfeed, so I could never have a breast cancer […]”  M21, screener with primary education, ≥50 years |
| Perceived negative outcomes from mammography – Intrapersonal factors ranging from negative perceptions of the mammogram procedure to fear of the outcome from mammogram, hindered uptake. Majority of participants cited fear of pain from mammography as a reason for not screening. Hearing unpleasant experiences of pain and discomfort from friends created much apprehension about attending mammography. One participant did not attend repeat screening after a very painful experience. Another participant cited a bad after-experience from mammography, where she felt very anxious having to wait for a month for the mammogram result. Some participants viewed the breast as synonymous with being woman, and associated its loss with the loss of femininity. One participant recounted the fear of losing her breast kept her from attending a follow-up appointment at the hospital although a breast abnormality had been detected on her mammogram. One screener shared that some may be concerned over friends knowing of them going for mammography, and asking if there was something wrong with them. Some participants mentioned that community-level factor such as modesty concerns that their bodies should only be exposed to their husbands. | “Because I also scared, right, got cancer. Then.. I think better I check lah.. Ah.. then what, see.. the.. poly (polyclinic) also call me come down and check, I also don’t want right. Then at home, I do (check) myself lah.”  M18, non-screener with no formal education, ≥50 years  “Because last time right, like this like that.. my friend said very pain. Because they want to find ah, small ah. Give me money also I don’t want. Sakit [pain in Malay language] […]”  M45, non-screener with secondary education, ≥50 years  “Why they are doing all these to the patient […] It is hurting people right […] Not for me, not anymore. I don’t believe in this anymore.”  M38, screener with tertiary education, ≥50 years  “[…] it takes a month that time la, that time was a month the result then come out. Not so fast, so long you know [laughs]. Ya that time. A few, 10 years back ah is like that. 10 years you know. 1 month I cannot eat, because I feel so scared I don’t know what’s happening. Is it is it really cancer or what [laughs]. Cannot eat, cannot eat, just cry because it take one month.”  M27, screener with secondary education, 40-49 years  “[…] For women, in general, regardless what what culture whatever. Because breast is synonymous with women [laugh] right. So without it, the person feel less of a woman.”  M47, non-screener with tertiary education, ≥ 50 years  “[…] if anything happened, “Eh, where’s my breasts? I don’t have any breast”. You know. […] They [referring to her children] ask they ask.. but I still don’t want to go. I say you go with me also, it’s my body, not your body. The doctor would check me, and then I have to go.. (for) further investigation. […] I cried. Scared la.”  M21, screener with secondary education, ≥50 years  “[…] Because they scare(d) later.. ah I got… like that.. ah… they scare(d) like (to) be the talk of the town.”  M10, screener with secondary education, ≥50 years  “Yeah, because you know, in Islam, right, for those who are really staunch Muslims right, they.. don’t want anyone else other than their husbands.. to touch them. […]”  M29, non-screener with tertiary education, 40-49 years |
| Perceived costs of screening – These included intrapersonal factors such as finance, interpersonal factors such as family responsibilities, and environmental factors such as inconvenience. A few women cited cost as a reason for not undergoing mammography. Some did not know that the compulsory MediSave can be used to pay for mammography for women aged 50 and above. Concerns over expensive treatment bills was reported by another participant as a reason for some not to attend screening. Some women expressed their intention to attend mammography but time constraints, family commitments of having to take care of grandchildren, and work commitments hindered them from attending mammography. The hassle of having to go to a hospital to make an appointment and long waiting time for a mammogram was another reason given by a non-screener with tertiary education. She was unaware that mammography was available at polyclinics where appointment could be made without doctor’s recommendation. | “[…] Then, for me.. so difficult. I cannot afford, I cannot force down “You must give me money, I want to go check-up”. Cannot lah. Because my daughter also got no money.[…]  M40, non-screener with secondary education, ≥ 50 years  “Oh you can use off the MediSave [looking at the Health Promotion Board’s website] oh okay, then I’m worried- I’m not worried, I have MediSave. I didn’t know MediSave can use […]”  M29, non-screener with tertiary education, 40-49 years  “[…] Because, my family like this ah, sometimes got money, sometimes no money. Then how, if I go to hospital, how? Must pay, must- if I go operation ah.. ah.. if 100 percent (success rate) okay. If 50-50-, how? Ah. Then the operation also must pay.”  M40, non-screener with secondary education, ≥ 50 years  “[…] Some no time to go. I think like myself lah, work work work work. Then ah, when she remind me last week, oh yay a ya, sorry. Okay I go I go […]”  M14, screener with secondary education, ≥ 50 years  “Hm.. as what I mentioned, I- I did try to.. go, but, you know. […] doctor need to order for you, need to follow-up with you, you need to erm.. you know, erm.. ah, the ordering, its.. it’s a.. it’s a trouble lah. Aiyoh [expression of exasperation], why must go through all the trouble just to ask for, you know, this screen.[..]”  M20, non-screener with secondary education, 40-49 years |
| Misinformation on breast cancer and mammogram – Two non-screeners, with diploma education, expressed fear of mammogram triggering cancer cells to spread. Specifically, radiation exposure and squeezing of the breast during mammography were perceived to trigger existing cancer cells in one’s body to spread. One married woman with 10 years of schooling believed breast cancer was caused by breast milk ‘clogging up’ in the breast. Some participants did not see a need for mammography because they feel well. They also believed that breast cancer cannot be treated even if detected early, and that they were too old for screening and subsequent treatments. | “[…] I’m actually kind of being advised (by family) not to do it. The reason is because as I mentioned, that the- the cancer cell is already in us. It’s just that for some reason, some people actually believe that when you do mammogram, will actually trigger the cancer cells to actually become active. So I’m not sure how true is that, but, erm.. I never thought of going through.. mammogram.”  M48, non-screener with tertiary education, 40-49 years  “[…] I, I never, I never give birth. And then no, no milk. That’s why maybe no problem lah. Maybe I think like that. Very soft. Some people got ever give birth, and then ah, breast, the milk never come out. Become cancer?”  M52, non-screener with secondary education, ≥ 50 years  “Ah I always check (breast self-examination) […] Maybe like pimples like that. Little bit pimples, not pain, okay lah. […] No pain, okay ah, I see first lah. If let it go, if can cure, cure lah. If pain gone okay. If still pain, ah, sure go.”  M52, non-screener with secondary education, ≥ 50 years |
| Religious belief – A non-screener shared on the will of God in controlling illness whereby some may perceive that if one was destined to have breast cancer, and one should accept one’s fate. Being able to depart from this world with a complete body as given by God, rather than having one’s breast removed because of breast cancer, was reported by her as a potential reason that others do not attend screening. | “[…] like to them, it’s fated, ok, it’s fated, so they accept the fate that they having- they are having erm cancer, cancer of the breast […] certain people think that it’s- it’s erm, you know they say that they would rather go as- like proper lah, one full body rather than oh no, oh I-I remove, I- I left with either right breast or either left breast, so they might think that it’s not complete lah.”  M20, non-screener with secondary education, 40-49 years |
| Distrust – Some participants shared general distrust of doctors. | “For me, I don’t trust. Because my dad take Panadol overdose, die. From that incident, from the age of 40, I don’t believe in doctors, I don’t know but I won’t go.”  M38, screener with tertiary education, ≥ 50 years |
| Strategies to overcome psychological barriers  Modesty concern – There is no need to be shy because many women do so too. Staff performing the mammography is always a woman as well. | “[…] Because so many people do, how come she want to spread here here [spread the news on one going for mammogram]? No ah.. Don’t be shy, for your own good.”  M23, screener with secondary education, ≥ 50 years |
| Fear of pain – Relax and use self-talk to convince self that the pain is for few minutes only and disappears when they take the machine off. | “[…] A bit awhile only pain. “Pak [sound of mammogram]” Like you press lah, sure pain lah. After that okay already. For about 5 to 10 seconds only. Okay already.”  M16, screener with secondary education, ≥ 50 years |
